# LncRNA LINC00998 inhibits the malignant glioma phenotype via the CBX3-mediated c-Met/Akt/mTOR axis

**DOI:** 10.1038/s41419-020-03247-6

**Published:** 2020-12-02

**Authors:** Haiping Cai, Yanjiao Yu, Xiangrong Ni, Cong Li, Yuanjun Hu, Jing Wang, Furong Chen, Shaoyan Xi, Zhongping Chen

**Affiliations:** 1 Department of Neurosurgery/Neuro-oncology, Sun Yat-sen University Cancer Center, State Key Laboratory of Oncology in South China, Collaborative Innovation Center for Cancer Medicine, Guangzhou, 510060 PR China; 2 Department of Pathology, Sun Yat-sen University Cancer Center, State Key Laboratory of Oncology in South China, Collaborative Innovation Center for Cancer Medicine, Guangzhou, 510060 PR China

**Keywords:** Cancer therapy, CNS cancer

## Abstract

Long noncoding RNAs (lncRNAs), once considered to be nonfunctional relics of evolution, are emerging as essential genes in tumor progression. However, the function and underlying mechanisms of lncRNAs in glioma remain unclear. This study aimed to investigate the role of LINC00998 in glioma progression. Through screening using TCGA database, we found that LINC00998 was downregulated in glioblastoma tissues and that low expression of LINC00998 was associated with poor prognosis. Overexpression of LINC00998 inhibited glioma cell proliferation in vitro and in vivo and blocked the G1/S cell cycle transition, which exerted a tumor-suppressive effect on glioma progression. Mechanistically, RNA pull-down and mass spectrometry results showed an interaction between LINC00998 and CBX3. IP assays demonstrated that LINC00998 could stabilize CBX3 and prevent its ubiquitination degradation. GSEA indicated that LINC00998 could regulate the c-Met/Akt/mTOR signaling pathway, which was further confirmed by a rescue assay using siRNA-mediated knockdown of CBX3 and the Akt inhibitor MK2206. In addition, dual-luciferase assays showed that miR-34c-5p could directly bind to LINC00998 and downregulate its expression. Our results identified LINC00998 as a novel tumor suppressor in glioma, and LINC00998 could be a novel prognostic biomarker, providing a strategy for precision therapy in glioma patients.

## Introduction

Glioma is the most common and malignant form of primary central nervous tumor in adults, accounting for more than 50% of all primary brain tumors^1^. The average survival of patients with glioblastoma is 12–15 months due to the high invasion and proliferation rates^2^. The current clinical treatment of glioma includes surgery, chemotherapy, radiotherapy, targeted therapy, and immunotherapy. However, the prognosis of glioma remains unfavorable with a high recurrence rate after initial treatment^3^. Thus, identifying new targets for therapeutic intervention and developing novel diagnostic approaches greatly needed for early diagnosis and intervention for glioblastoma.

Accumulating evidence has demonstrated that long noncoding RNAs (lncRNAs) are involved in tumor progression and have been identified as biomarkers for tumor diagnosis, treatment, and prognosis in recent years^4–6^. LncRNAs are functional RNA segments longer than 200 nucleotides that used to be considered redundant transcriptional products^7^. LncRNAs play an important role in the regulation of numerous cellular processes, including cell proliferation, cell cycle, apoptosis, and differentiation^7–11^. Many lncRNAs have been well characterized in tumor progression. For example, LINC00336 acts as an endogenous sponge of microRNA 6852 (MIR6852) to inhibit ferroptosis in lung cancer^12^. Tumor-associated macrophage-derived extracellular vesicle-packaged HIF-1α-stabilizing lncRNA enhances aerobic glycolysis and apoptotic resistance in breast cancer cells^13^. Xist RNA has a tumor-suppressing function in hematologic cancer^14^. Intriguingly, the expression of lncRNAs is significantly tissue specific compared with that of protein-coding genes^15,16^. Aberrant expression signatures of lncRNAs have been revealed to be correlated with glioma development and malignant progression^17,18^. We searched for differential gene expression between glioblastoma tissues and paired adjacent normal tissues from The Cancer Genome Atlas (TCGA) and determined that LINC00998 was downregulated in glioblastoma, which implied a tumor-suppressing role of LINC00998 in glioblastoma progression. However, the function of LINC00998 in glioma remains unclear. The goal of this study was to explore the molecular mechanisms and effects of LINC00998 on the biological behaviors of glioma cells and provide promising therapeutic targets in human glioma.

Here, our data indicate that downregulation of LINC00998 is correlated with poor prognosis in glioma patients. LINC00998 downregulation promoted malignant phenotypes of glioma cells. Mechanistically, LINC00998 prevented CBX3 ubiquitination and promoted tri-methylation of histone H3K9 in c-Met promoter region, further downregulated the c-Met/Akt/mTOR signaling pathway, and then attenuated glioma progression. The tumor-suppressive function and mechanisms of LINC00998 in glioma were characterized.

## Results

### LINC00998 is downregulated in glioma and associated with poor survival

**Fig. 1:**
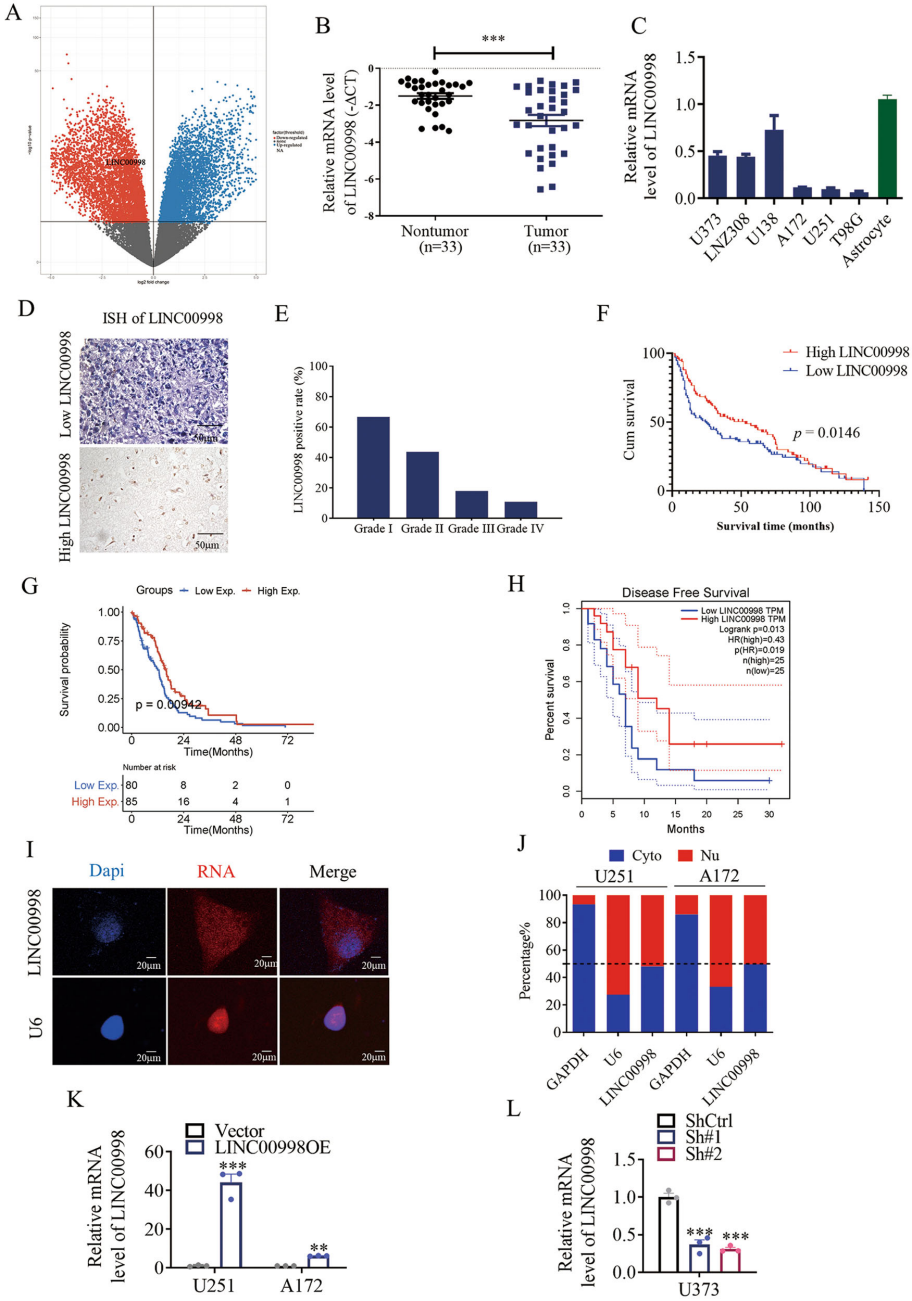
LINC00998 is downregulated in glioma and associated with poor survival. **a** Volcano plot analysis of differentially expressed genes between glioblastoma tissues and paired adjacent normal tissues from TCGA showing that LINC00998 was downregulated in glioblastoma tissues. **b** mRNA levels of LINC00998 were detected by qRT-PCR in glioma tissues and paired adjacent normal tissues from the SYSUCC cohort (*n* = 33). **c** mRNA levels of LINC00998 were detected by qRT-PCR in six glioma cell lines and normal immortalized astrocytes. **d** ISH results of LINC00998 expression. (up: low LINC00998 expression; down: high LINC00998 expression) scar bar = 50 μm. **e** LINC00998 positive rate of grades I–IV glioma (*n* = 12, 81, 72, and 64, respectively) according to the ISH results on a tissue array. **f** Kaplan–Meier analysis showed that downregulation of LINC00998 predicted poor overall survival in the SYSUCC cohort (*n* = 229, *P* = 0.0146). **g**, **h** Kaplan–Meier analysis showed that downregulation of LINC00998 predicted poor overall survival and disease-free survival in the TCGA cohort (*n* = 165, *P* = 0.00942 and *n* = 50, *P* = 0.013, respectively). **i** Fluorescence in situ hybridization assay revealed that LINC00998 was located both in the nucleus and cytoplasm. **j** Subcellular fractionation analysis confirmed that LINC00998 was located both in the nucleus and cytoplasm. **k**, **l** Overexpression and knockdown of LINC00998 in A172, U251 and U373 cells was confirmed by qRT-PCR (*P < 0.05, **P < 0.01, ***P < 0.001).

To identify lncRNAs that significantly affect glioma progression, we first obtained lncRNAs that were differentially expressed between glioblastoma tissues and paired adjacent normal tissues from TCGA database. Then, we sorted these lncRNAs according to the log2 fold change and found that LINC00998 was expressed at lower levels in glioblastoma tissues than in paired adjacent normal tissues (Fig. 1a), suggesting that it might play a role in glioblastoma progression. Bioinformatics analysis showed that LINC00998 is located on chromosome 7q31.1, and the secondary structure of LINC00998 is presented in Supplementary Fig. S1A. According to the coding potential calculator, the coding potential of LINC00998 is very low (Supplementary Fig. S1B). We then detected the expression of LINC00998 in 33 primary glioma samples and their corresponding nontumor tissues, which showed that LINC00998 was significantly downregulated in tumor samples (Fig. 1b). In addition, LINC00998 was also detected in six glioma cell lines and a normal astrocyte cell line by qRT-PCR. We found that LINC00998 was significantly downregulated in glioma cell lines compared to normal astrocytes (Fig. 1c). A tissue microarray containing 229 patient samples (12, 81, 72, and 64 cases of grades I–IV glioma, respectively) was applied for in situ hybridization (ISH). We found that the positive rate of LINC00998 was significantly lower in high-grade glioma (III and IV glioma) than in low-grade glioma (I and II glioma) (Fig. 1d, e). Furthermore, the lower level of LINC00998 was closely associated with poorer overall survival (OS) (Fig. 1f), which was in accordance with the data from TCGA that the lower level of LINC00998 correlated with poorer OS and disease-free survival in TCGA datasheet (*n* = 165, *P* = 0.00942 and *n* = 50, *P* = 0.013, respectively; Fig. 1g, h). To determine the subcellular localization of LINC00998, fluorescence in situ hybridization (FISH) assay and subcellular fractionation analysis were performed. The results showed that LINC00998 was located in both the nucleus and cytoplasm (Fig. 1i, j). Collectively, our results showed that LINC00998 was downregulated in glioma and that low LINC00998 expression was associated with poorer survival.

### LINC00998 impairs glioma proliferation in vitro and in vivo

**Fig. 2:**
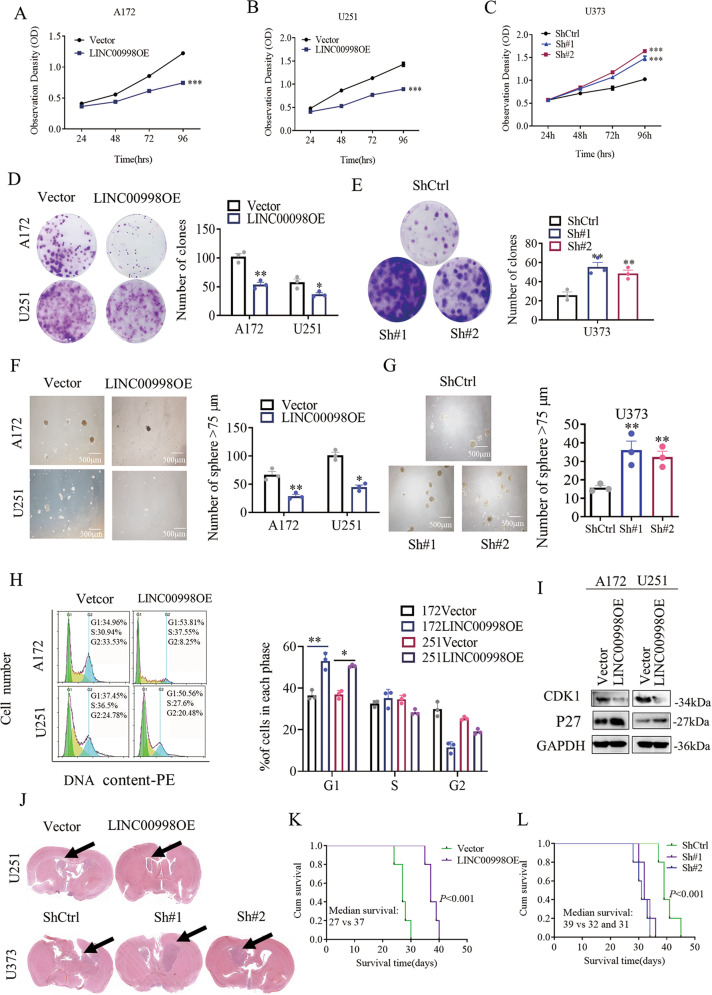
LINC00998 impairs glioma proliferation in vitro and in vivo. **a**, **b** Overexpression of LINC00998 inhibited A172 and U251 growth. **c** Knockdown of LINC00998 promoted U373 growth. **d** Overexpression of LINC00998 inhibited A172 and U251 clone formation ability. **e** knockdown of LINC00998 promoted U373 clone formation ability. **f** Overexpression of LINC00998 inhibited A172 and U251 sphere formation ability. **g** Knockdown of LINC00998 promoted U373 sphere formation ability. **h** Overexpression of LINC00998 blocked the cell cycle transition at G1/S. **i** Overexpression of LINC00998 decreased CDK1 expression and increased P27 protein expression. **j** H&E staining of brain sections demonstrated a significant decrease or increase in tumor volume after LINC00998 overexpression in U251 cells or knockdown in U373 cells, respectively, at 25 days post implantation (black arrows indicate tumor location). **k, l** Kaplan–Meier survival curves show a significant increase in median survival of U251 LINC00998OE tumor-bearing mice and decrease in median survival of both U373 sh#1 and sh#2 tumor-bearing mice. Scale bar = 500 μm (*P < 0.05, **P < 0.01, ***P < 0.001).

Because LINC00998 was downregulated in both glioma cells and tissues, we further investigated the role of LINC00998 in glioma cell behaviors. According to the mRNA level of LINC00998 in six glioma cell lines, LINC00998 was overexpressed in A172 and U251 cells, and it was knocked down in U373 glioma cells (Fig. 1k, l). Cell proliferation assays showed that overexpression of LINC00998 impaired A172 and U251 cell growth, while knockdown of LINC00998 promoted U373 growth (Fig. 2a–c). Furthermore, clone formation ability and sphere formation ability were inhibited after overexpression of LINC00998 in A172 and U251 cells, while knockdown of LINC00998 promoted U373 clone formation ability and sphere formation ability (Fig. 2d–g). Further, we investigated whether LINC00998 induced apoptotic cell death in glioma and found that overexpression of LINC00998 increased annexin V positive cell rates in both A172 and U251 glioma cells, while knockdown of LINC00998 decreased annexin V positive cells in U373 (Supplementary Fig. S2). Overexpression of LINC00998 blocked the G1/S cell cycle transition (Fig. 2h). We also detected key cell cycle proteins, and the results showed that overexpression of LINC00998 decreased CDK1 expression and increased P27 protein expression (Fig. 2i). These results implied that LINC00998 might regulate CDK1 and P27 expression to promote glioma cell proliferation. Since LINC00998 inhibited glioma cell proliferation in vitro, the tumor-suppressive role of LINC00998 in vivo was also investigated. Empty vector-transfected cells, LINC00998-transfected U251 cells, and LINC00998-knockdown U373 cells (named Sh#1 and Sh#2) were intracranially injected into BALB/c nu mice. The results showed that mice from the LINC00998-transfected group survived longer than mice from the empty vector group in U251 cells. In contrast, mice from the LINC00998-knockdown group had shorter survival than mice from the ShCtrl group in U373 cells. Thus, these findings indicated that LINC00998 impairs glioma proliferation in vitro and in vivo.

### LINC00998 associates with CBX3 and prevents CBX3 ubiquitination degradation

**Fig. 3:**
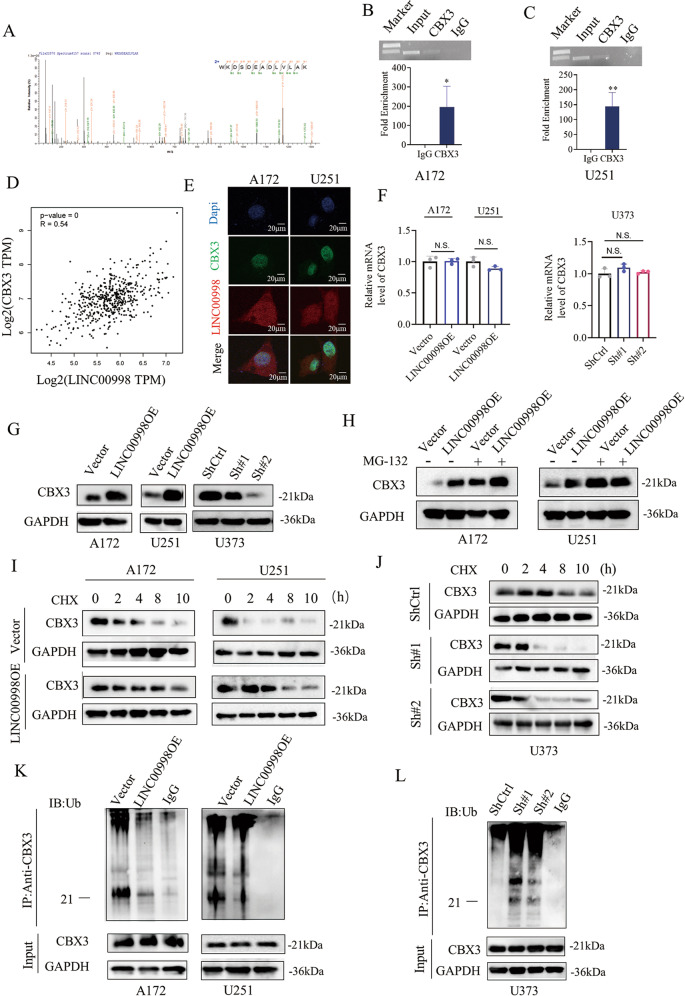
LINC00998 associates with CBX3 and prevents CBX3 ubiquitination degradation. **a** Mass spectrometry analysis of CBX3 binding to LINC00998. **b**, **c** RNA immunoprecipitation assay in A172 and U251 cells confirmed the interaction of CBX3 and LINC00998. **d** Coexpression analysis showed a strong correlation between CBX3 and LINC00998 in TCGA database (*R* = 0.54). **e** Fluorescence in situ hybridization combined with immunofluorescence assays demonstrated the colocalization of LINC00998 and CBX3 in the cell nucleus. **f** mRNA levels of CBX3 were not changed after LINC00998 overexpression in A172 and U251 cells or knockdown in U373 cells, as detected by qRT-PCR. **g** Protein expression of CBX3 increased or decreased after LINC00998 overexpression in A172 and U251 cells or knockdown in U373 cells, respectively. **h** CBX3 downregulation by LINC00998 overexpression was reversed by treatment with the proteinase inhibitor MG-132 (5 µM, 24 h). **i**, **j** Western blotting results showed that LINC00998 overexpression stabilized CBX3 protein, while LINC00998 knockdown shortened the half-life of CBX3 after CHX treatment at 20 µg/ml for 0, 2, 4, 8, and 10 h. **k**, **l** IP assays showed that LINC00998 overexpression decreased CBX3 ubiquitination levels, while LINC00998 knockdown increased ubiquitination levels. (*P < 0.05, **P < 0.01, ***P < 0.001).

Accumulating evidence has revealed that lncRNAs can bind to functional proteins and affect tumor cell behavior. We performed RNA pull-down assays and mass spectrometry analysis to investigate proteins that could bind to LINC00998. Among the LINC00998 pull-down proteins, chromobox 3 (CBX3) was one of the most abundant proteins (Fig. 3a and Supplementary Table S2). Then, an RNA immunoprecipitation (RIP) assay was performed to validate the interaction of LINC00998 and CBX3 protein (Fig. 3b, c). Coexpression analysis of LINC00998 and CBX3 also showed a strong correlation in TCGA database (*R* = 0.54, Fig. 3d). Furthermore, RNA FISH combined with immunofluorescence assays confirmed the colocalization of LINC00998 and CBX3 in the cell nucleus (Fig. 3e). Subsequently, we detected the mRNA level of CBX3 in A172, U251, and U373 cells after LINC00998 overexpression or knockdown. qRT-PCR results showed that the mRNA level of CBX3 did not change regardless of the overexpression or knockdown of LINC00998 in A172, U251, and U373 cells (Fig. 3f). Protein expression of CBX3 was also detected in the three cell lines after LINC00998 overexpression or knockdown. Western blotting results showed that overexpression of LINC00998 increased CBX3 protein levels, while knockdown of LINC00998 decreased CBX3 protein levels (Fig. 3g). These results indicated that LINC00998 might regulate CBX3 protein levels through protein posttranslational modification (PTM). Therefore, A172 and U251 cells were treated with the proteasomal inhibitor MG-132 for 24 h, and the results showed that the CBX3 protein level could be restored by MG-132 (Fig. 3h). In addition, overexpression of LINC00998 had marked effects on CBX3 stabilization in A172 and U251 cells, prolonging the half-life of CBX3, while knockdown of LINC00998 shortened the half-life of CBX3 in U373 cells (Fig. 3i, j). Moreover, we carried out IP assays in the three glioma cell lines using an anti-CBX3 antibody and detected the ubiquitin levels of CBX3 by western blotting. As shown, overexpression of LINC00998 significantly decreased the levels of ubiquitinated CBX3, while knockdown of LINC00998 increased the levels of ubiquitinated CBX3 (Fig. 3k, l). Collectively, these results demonstrated that LINC00998 bound to CBX3 and stabilized CBX3 by preventing its ubiquitination.

### LINC00998 promotes tri-methylation of histone H3K9 in c-Met promoter region and further attenuates the c-Met/Akt/mTOR signaling pathway

**Fig. 4:**
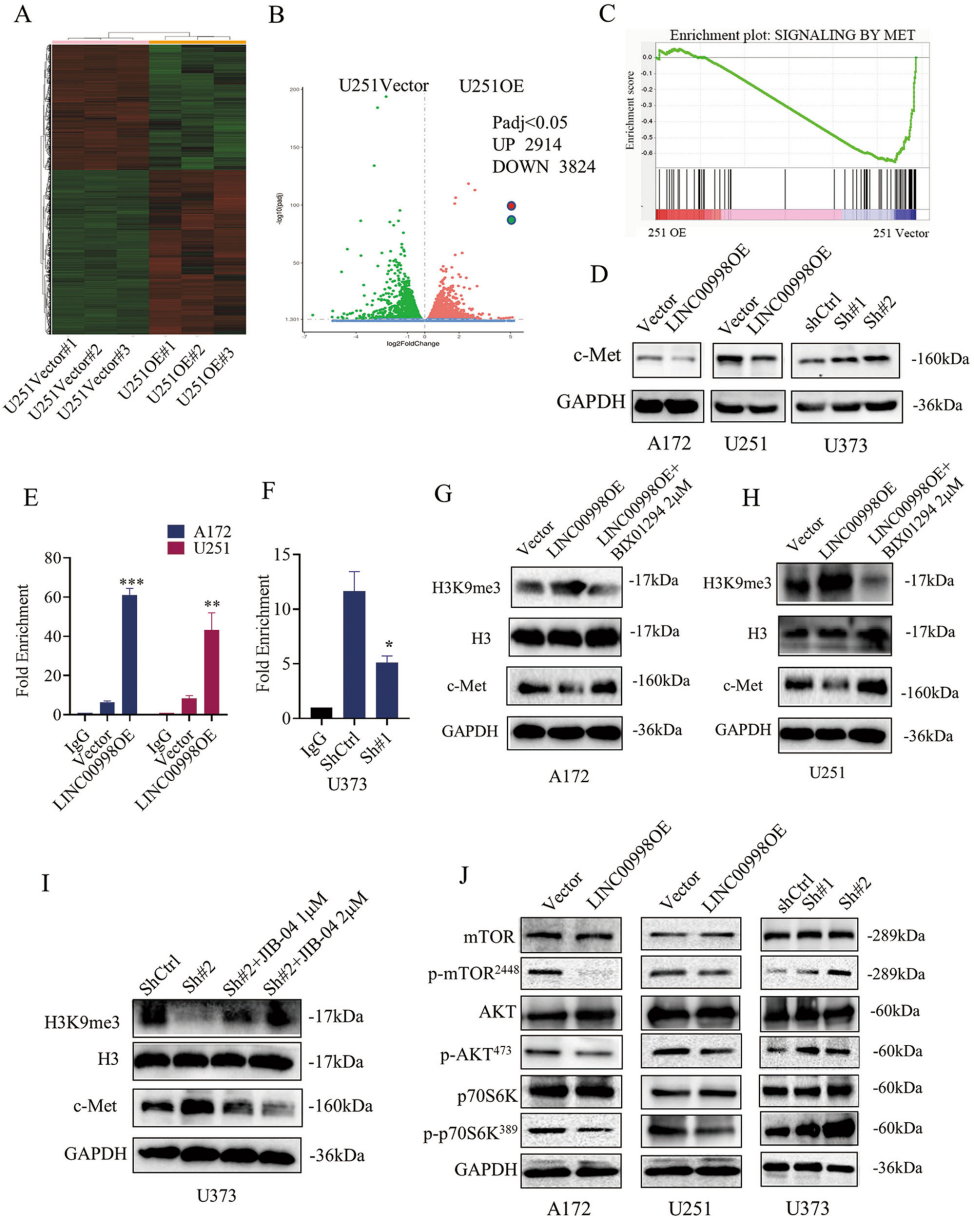
LINC00998 promotes tri-methylation of histone H3K9 in c-Met promoter region and inhibits the c-Met/Akt/mTOR signaling pathway. **a** RNA sequencing analysis of the U251 vector and U251 LINC00998 overexpression groups. **b** Volcano plot analysis showed that 2914 genes were upregulated, while 3824 genes were downregulated in the U251 OE group. **c** GSEA of differentially expressed genes showed that overexpression of LINC00998 might regulate the c-Met signaling pathway. **d** Western blotting results confirmed that overexpression of LINC00998 downregulated c-Met expression, while knockdown of LINC00998 increased c-Met expression. **e** ChIP assay confirmed that overexpression of LINC00998 increased tri-methylation of histone H3K9 in c-Met promoter region compared to the vector group. Anti-H3K9me3 and anti-IgG antibodies were used. **f** knockdown of LINC00998 decreased tri-methylation of histone H3K9 in c-Met promoter region compared to the ShCtrl group. Anti-H3K9me3 and anti-IgG antibodies were used. **g**, **h** Western blotting results showed that BIX01294 (2 μM for 24 h) could decreased H3K9me3 level and increased protein level of c-Met. **i** Western blotting results showed that JIB-04 treatment (1 or 2 μM for 24 h, respectively) could increase H3K9me3 level and decreased protein level of c-Met. **j** Phosphorylation of key c-Met/Akt/mTOR signaling pathway proteins was decreased or increased after LINC00998 overexpression or knockdown, respectively. (*P < 0.05, **P < 0.01, ***P < 0.001).

To determine the signaling pathway affected by LINC00998 in impairing glioma growth, RNA sequencing was performed in two groups (U251 vector and U251 LINC00998 overexpression (U251 OE)). The results showed that 2914 genes were upregulated, while 3824 genes were downregulated in the U251 OE group compared to the U251 vector group based on log2 fold change (Fig. 4a, b). GSEA pathway analysis was performed on the differentially expressed genes, and the results showed that overexpression of LINC00998 could regulated the c-Met signaling pathway, which plays an important role in cell proliferation (Fig. 4c). Western blotting assay confirmed that overexpression of LINC00998 increased protein level of c-Met, while knockdown of LINC00998 decreased protein level of c-Met (Fig. 4d). As reported that CBX3 could Interact with histone H3 methylated at “Lys-9,”^19^ we investigated whether LINC00998 downregulated protein level of c-Met by stabilized CBX3 and promoted tri-methylation of histone H3K9 in c-Met promoter region. Chromatin immunoprecipitation (ChIP) assay was performed and in chromatin fractions that were pulled down by anti-H3K9me3 antibody, qRT-PCR results showed that c-Met level in LINC00998OE group was more than that in vector group, while knockdown of LINC00998 had the opposite effect, which implied that overexpression of LINC00998 increased tri-methylation of histone H3K9 in c-Met promoter region (Fig. 4e, f). Western blotting and Immunofluorescence assays also showed the similar results that overexpression of LINC00998 increased tri-methylation of histone H3K9 (Supplementary Fig. S3A, B). Furthermore, in order to investigate the effect of histone H3K9 tri-methylation level in c-Met promoter region, a histone methyltransferase inhibitor BIX01294 and a histone demethylase inhibitor JIB-04 were used. Glioma cells A172 and U251 in LINC00998OE group were treated with BIX01294 at 2 μM for 24 h and their protein level of H3K9me3 and c-Met were detected. Results showed that BIX01294 decreased H3K9me3 level and increased protein level of c-Met (Fig. 4g, h). On the contrary, glioma cells U373 in LINC00998Sh#2 group were treated with JIB-04 at 1 or 2 μM for 24 h, respectively. Results showed that JIB-04 treatment could increase H3K9me3 level and decreased protein level of c-Met (Fig. 4i). Since Akt and mTOR have been reported to be key downstream genes of c-Met, we detected the protein expression of c-Met/Akt/mTOR pathway members and their phosphorylation levels. Western blotting results showed that overexpression of LINC00998 downregulated c-Met protein levels and inhibited Akt, mTOR, and p70S6K phosphorylation levels in A172 and U251 cells, while knockdown of LINC00998 had the opposite effect in U373 cells (Fig. 4j). Collectively, these results indicated that LINC00998 promoted tri-methylation of histone H3K9 in c-Met promoter region and inhibited the c-Met/Akt/mTOR signaling pathway.

### Knockdown of CBX3 reverses the tumor growth inhibitory effect of LINC00998

**Fig. 5:**
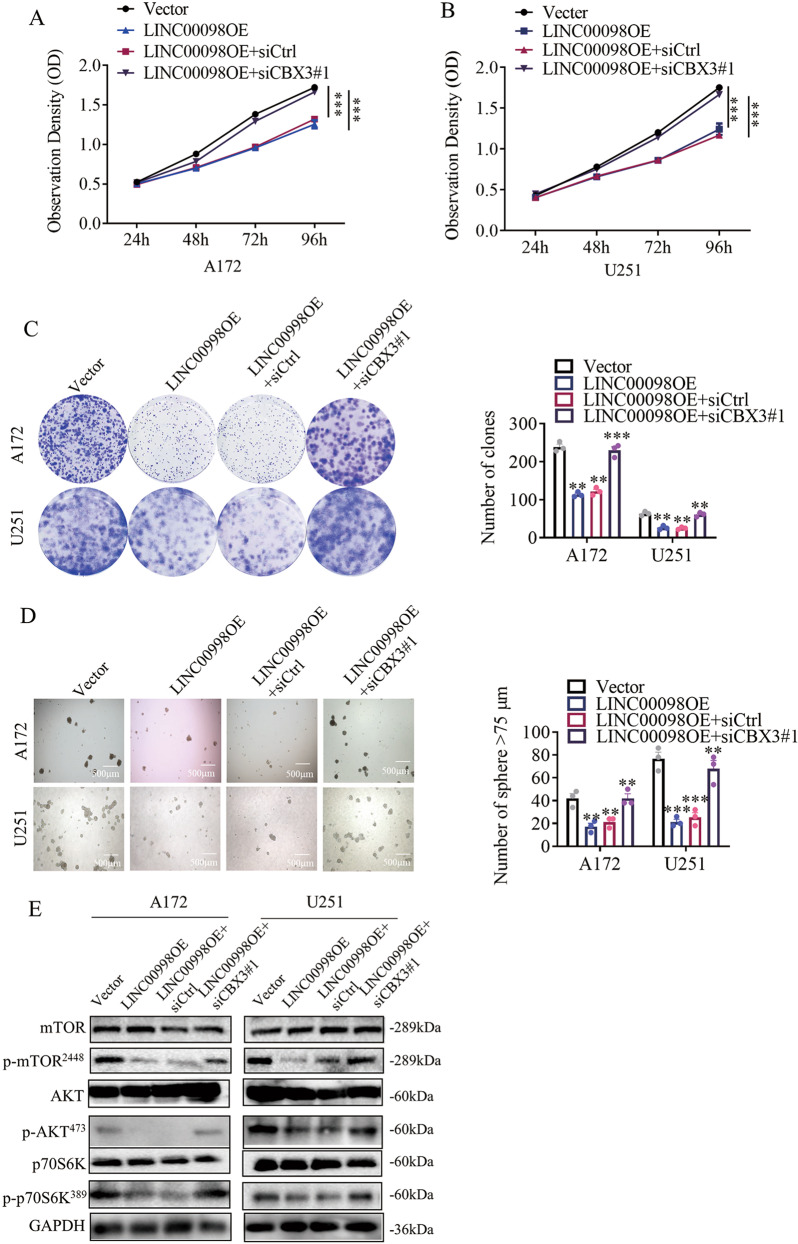
Knockdown of CBX3 reversed the tumor growth inhibitory effect of LINC00998. **a, b** Overexpression of LINC00998 inhibited A172 and U251 cell growth, while siRNA-mediated knockdown of CBX3 reversed the growth inhibitory effect of LINC00998. **c** Overexpression of LINC00998 inhibited A172 and U251 cell clone formation ability, while siRNA-mediated knockdown of CBX3 reversed the growth inhibitory effect of LINC00998. **d** Overexpression of LINC00998 inhibited A172 and U251 cell sphere formation ability, while siRNA-mediated knockdown of CBX3 reversed the growth inhibitory effect of LINC00998. **e** Overexpression of LINC00998 decreased the phosphorylation levels of Akt, mTOR, and P70S6K, while siRNA-mediated knockdown of CBX3 increased the phosphorylation levels of Akt, mTOR and P70S6K. (*P < 0.05, **P < 0.01, ***P < 0.001).

Since LINC00998 binds to CBX3 and prevents CBX3 ubiquitination degradation, we investigated whether knockdown of CBX3 could reverse the LINC00998 overexpression-mediated suppression of malignant phenotypes of glioma cells. First, we detected the growth ability of A172 and U251 cells under different conditions. We found that the growth of both A172 and U251 cells was inhibited with LINC00998 overexpression, while the inhibitory phenotypes were reversed after treatment with siRNA targeting CBX3 (Fig. 5a, b). Similarly, the clone formation and sphere formation abilities of A172 and U251 cells were both impaired in the LINC00998 overexpression group but reversed in the siCBX3#1 group (Fig. 5c, d). Because overexpression of LINC00998 inhibited the c-Met/Akt/mTOR signaling pathway, we detected the phosphorylation levels of key proteins after siCBX3 treatment in A172 and U251 cells. As expected, overexpression of LINC00998 inhibited the phosphorylation levels of mTOR, Akt, and p70S6K. However, the phosphorylation levels of these key signaling pathway proteins were restored after siCBX3 treatment (Fig. 5e), which indicated that CBX3 might be essential for LINC00998 function. Collectively, these results demonstrated that knockdown of CBX3 reversed LINC00998 overexpression-mediated suppression of malignant phenotypes of glioma cells and Akt/mTOR signaling pathway key protein phosphorylation levels.

### Akt inhibitor exerts a similar effect as LINC00998 on glioma cells

**Fig. 6:**
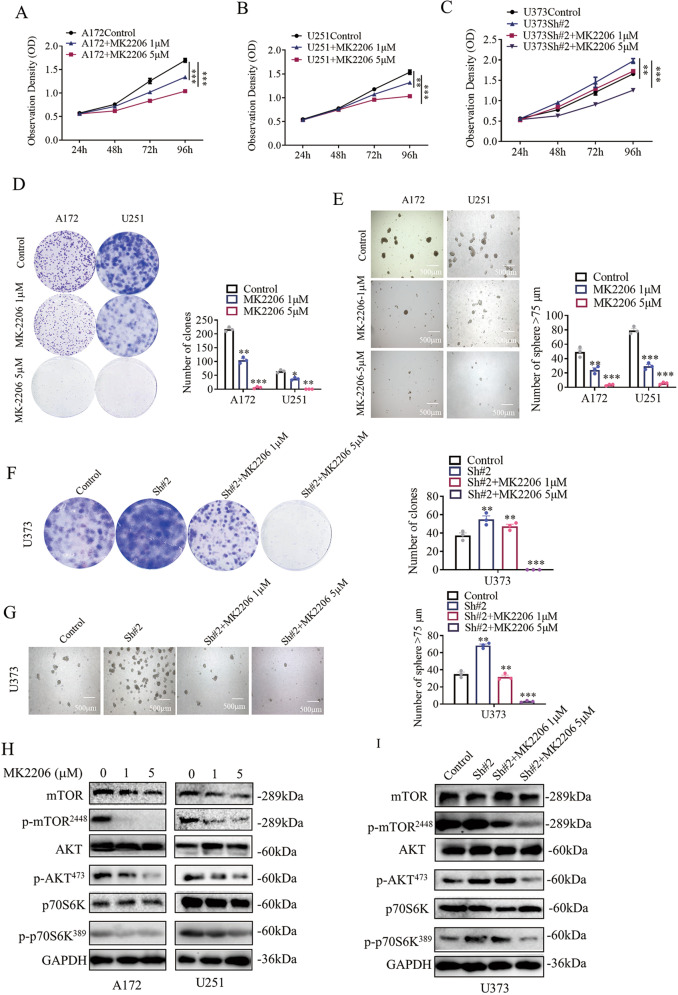
Akt inhibitor exerts a similar effect as LINC00998 on glioma cells. **a**, **b** A172 and U251 cell growth was inhibited gradually in a dose-dependent manner after MK2206 treatment at 1 and 5 µM. **c** The LINC00998 knockdown-promoted growth of U373 cells was reversed by MK2206 treatment at both 1 and 5 µM. **d, e** Clone formation ability and sphere formation ability of A172 and U251 cells were inhibited gradually in a dose-dependent manner after MK2206 treatment at 1 and 5 µM. **f**, **g** The LINC00998 knockdown-promoted clone formation ability and sphere formation ability of U373 cells was reversed by MK2206 treatment at both 1 and 5 µM. **h** Phosphorylation levels of Akt, mTOR, and P70S6K in A172 and U251 cells were decreased after MK2206 treatment. **i** Phosphorylation levels of Akt, mTOR, and P70S6K in U373 cells with LINC00998 knockdown were reversed by MK2206 treatment at both 1 and 5 µM. (*P < 0.05, **P < 0.01, ***P < 0.001).

Since overexpression of LINC00998 inhibited c-Met/Akt/mTOR signaling pathway protein phosphorylation levels, the Akt inhibitor MK2206 was introduced into in vitro functional assays in A172, U251, and U373 cells. In our study, cell growth was impaired gradually in a dose-dependent manner in A172 and U251 cells upon exposure to MK2206 at 0, 1, and 5 µM for 24 h (Fig. 6a, b). The LINC00998 knockdown-promoted growth of U373 cells was reversed by MK2206 treatment at both 1 and 5 µM (Fig. 6c). The clone formation and sphere formation abilities of A172 and U251 cells were both inhibited when the cells were exposed to MK2206 (Fig. 6d, e). MK2206 treatment also rescued the LINC00998 knockdown-promoted clone formation and sphere formation ability of U373 cells (Fig. 6f, g). Then, we detected the phosphorylation of mTOR, Akt and p70S6K. Western blotting results showed that MK2206 decreased the phosphorylation of these key c-Met/Akt/mTOR signaling pathway proteins (Fig. 6h). In addition, the increased phosphorylation of mTOR, Akt, and p70S6K by knockdown of LINC00998 in U373 cells could be decreased after MK2206 treatment (Fig. 6i). Besides, upon the treatment of inhibitors for c-Met and mTOR (SU11274 and rapamycin), glioma cells (A172, U251, and U373) exhibited a similar effect as cells treated by LINC00998 (Supplementary Figs. S4 and S5). These findings suggested that LINC00998 inhibited the malignant glioma phenotype by decreasing the phosphorylation level of c-Met/Akt/mTOR signaling pathway proteins.

### LINC00998 is downregulated by miR-34c-5p

**Fig. 7:**
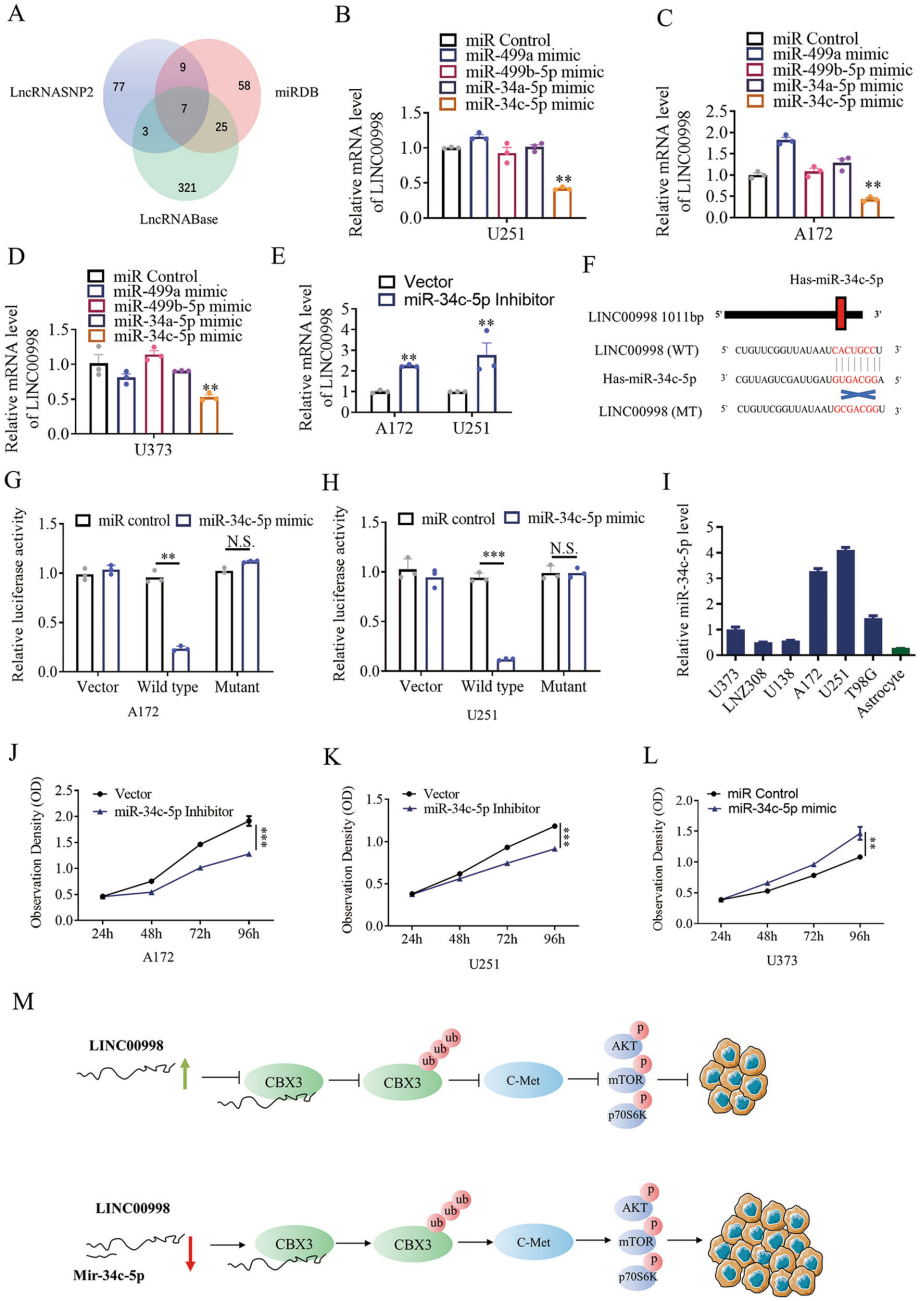
LINC00998 is downregulated by miR-34c-5p. **a** The bioinformatic databases LncRNASNP2, miRDB, and LncRNABase were used to identify potential miRNAs that could bind to LINC00998. **b–d** The mRNA levels of LINC00998 were downregulated by miR-34c-5p mimic in A172, U251, and U373 cells. **e** MiR-34c-5p inhibitor treatment upregulated LINC00998 expression in A172 and U251 cells. **f** A model describing the interaction between miR-34c-5p and LINC00998 and establishment of the WT-LINC00998 3ʹ UTR and MT-LINC00998 3ʹ UTR constructs. **g, h** Dual-luciferase reporter assay showed the relative luciferase activities in A172 and U251 cells after cotransfection of miR-34c-5p mimics or miR-control with WT-LINC00998-luc, MT-LINC00998-luc or empty vector with pRL-TK. **i** miR-34c-5p level were detected by qRT-PCR in six glioma cell lines and normal immortalized astrocytes. **j, k** miR-34c-5p inhibitor impaired A172 and U251 glioma cells growth. **l** miR-34c-5p mimic promoted U373 glioma cells growth. **m** A model was showed to describing the mechanism of how LINC00998 involved the regulation of malignant glioma phenotype (*P < 0.05, **P < 0.01, ***P < 0.001).

Recent studies have demonstrated that the interaction between lncRNAs and miRNAs is important for lncRNA regulation^20,21^. By exploring three bioinformatic databases, LncRNASNP2 (https://www.lncrnablog.com/), miRDB (http://mirdb.org/), and LncRNABase (https://www.lncrnablog.com), we found four miRNAs (miR-449a, miR-449b-5p, miR-34a-5p, miR-34c-5p) that could bind to LINC00998 (Fig. 7a). Then, we detected the LINC00998 mRNA level after miR-449a mimic, miR-449b-5p mimic, miR-34a-5p mimic, and miR-34c-5p mimic treatment in A172, U251, and U373 glioma cells. We found that only miR-34c-5p mimic treatment in A172, U251, and U373 downregulated LINC00998 mRNA levels compared to the control treatment (Fig. 7b–d). MiR-34c-5p inhibitor treatment in A172 and U251 cells increased LINC00998 mRNA levels in comparison to the control treatment (Fig. 7e), which implied an interaction between LINC00998 and miR-34c-5p. To further verify whether LINC00998 is a direct target of miR-34c-5p, we generated two constructs, wild-type (WT)-LINC00998 3ʹ UTR and mutant (MT)-LINC00998 3ʹ UTR. The empty luciferase reporter construct served as a control. A model describing the interaction between miR-34c-5p and LINC00998 and the establishment of the WT-LINC00998 3ʹ UTR and MT-LINC00998 3ʹ UTR constructs is shown in Fig. 7f. The relative luciferase activity in A172 and U251 cells was obviously decreased after cotransfection of miR-34c-5p mimic and WT-LINC00998-luc, while the luciferase activity was not affected in the MT-LINC00998-luc group and empty luciferase reporter construct group (Fig. 7g, h). We also detected the expression of miR-34c-5p on glioma and astrocyte cell line. MiR-34c-5p were upregulated in A172 and U251 but downregulated in U373 while maintained at lowest level in astrocytes (Fig. 7i). Furthermore, the proliferation of A172 and U251 was impaired by the treatment of miR-34c-5p inhibitor, which was even promoted in U373 treated by mimic of miR-34c-5p (Fig. 7j–l). Collectively, these data suggested that LINC00998 were downregulated by miR-34c-5p.

## Discussion

Glioma is the most common primary tumor in adults and has a high mortality rate. Although treatments including invasive resection, chemotherapy, and radiotherapy have improved the outcomes of glioma patients, the OS of these patients is still short. With the emerging identification of specific biomarkers that affect the biofunction of tumors, glioma genotype analysis can be more informative in providing a more accurate diagnosis, treatment, and prognosis for glioma management^22,23^. Accumulating evidence has demonstrated that lncRNAs play an essential role in glioma malignant progression, but the functional landscape of lncRNAs in human glioma progression remains unclear. In our study, we analyzed differential gene expression between glioblastoma tissues and paired normal tissues from TCGA database and found that LINC00998 was significantly downregulated in glioblastoma samples, which implied a tumor-suppressive function in glioma progression. Ye et al. also reported that LINC00998 expression was significantly lower in major depressive disorder (MDD) patients and associated with MDD^24^, but the function of LINC00998 in glioma remains unclear.

Accumulating evidence has revealed that different subcellular localizations of lncRNAs might indicate different functions and mechanisms. Our results showed that LINC00998 accumulated in both the nucleus and cytoplasm, which implied that it has a complex mechanism in inhibiting glioma progression. In addition, lncRNAs have been reported to bind to proteins and affect protein translational modifications or gene regulation by behaving as decoys for miRNA. We used RNA pull-down and mass spectrometry to investigate the proteins that bind to LINC00998, and the results showed that CBX3 was associated with LINC00998. CBX3 is located in the nucleus and binds to different nucleoproteins, which play different roles in cell progression. In addition, CBX3 was also reported to be located in the cytoplasm, interacting with actin in both cell compartments^25^. Report shows that histone H3K9 is methylated by SUV39H1 which creates a binding site for CBX3. SUV39H1 and CBX3 functionally interact to repress gene transcription^19^. The phosphorylation of CBX3 exerted a transcriptional repression effect on gene expression^26^. We detected the mRNA level of CBX3 after overexpression or knockdown of LINC00998 and found that the mRNA level of CBX3 did not change, while the protein level of CBX3 was increased or decreased, respectively. These results indicated a protein translational modification function of LINC00998. CBX3 levels are regulated at the both level of transcriptional and posttranslational. CBX3 is reported to be SUMOylated at Lys residues K5, K10, K21, K103, and K154^27–30^, which is PTMs of proteins. Treatment with the proteasomal inhibitor MG-132 or protein synthesis inhibitor cycloheximide (CHX) indicated that overexpression of LINC00998 stabilized CBX3 protein and prolonged its half-life. Further IP assays demonstrated that LINC00998 prevented CBX3 ubiquitination degradation, which was similar to other reports showing that lncRNAs have a protein translational modification function^31^. However, how LINC00998 inhibits ubiquitination of CBX3 still need to be dissected in detail. We will keep working to prove whether LINC00998 shifts its conformational structure and covers the recognition domain for ubiquitin proteins, or LINC00998 just serves as the scaffold to provide a platform for interaction between CBX3 and other proteins.

CBX3 can bind to histone H3 tails methylated at Lys-9 sites, implying a function as an inhibitor of gene expression^32–34^. Our study showed that LINC00998 promotes tri-methylation of histone H3K9 in c-Met promoter region through stabilized CBX3 and subsequently inhibited c-Met expression. Overexpression of LINC00998 promoted H3K9me3 level may attribute to the upregulation of CBX3 which stabilize H3K9me3 state. Furthermore, we confirmed that overexpression of LINC00998 decreased the phosphorylation levels of p-Akt^473^, p-mTOR^2448^, and p-p70S6K^389^, which are all key downstream proteins of c-Met. Knockdown of CBX3 could reverse the inhibitory phenotypes or phosphorylation levels of Akt, mTOR and p70S6K caused by LINC00998 overexpression, which implied that CBX3 might be an essential protein for LINC00998 function in glioma. In addition, small molecular inhibitor MK2206 reduced the malignant phenotype of glioma cells, similar to the effect of LINC00998 overexpression on glioma cells. Besides, we also confirmed that LINC00998 could be directly regulated by miR-34c-5p which is in accordance with the reports that miRNAs and lncRNAs interacts frequently^31^. A model was showed to describing the mechanism of how LINC00998 involved the regulation of malignant glioma phenotype (Fig. 7m).

In summary, our findings are the first to define LINC00998 as a tumor suppressor in glioma, and LINC00998 might serve as a potential prognostic biomarker for glioma patients. Knockdown of LINC00998 enables glioma cells to acquire high proliferation ability by regulating CBX3 and the c-Met/Akt/mTOR signaling pathway, which indicates that LINC00998 may provide a rationale for precision therapy in glioma patients.

## Materials and methods

### Clinical specimens

A total of 33 paired glioma samples together with corresponding nontumor tissues, and a tissue array (229 glioma samples) were obtained from glioma patients at Sun Yat-Sen University Cancer Center (SYSUCC) between 2001 and 2016 with written informed consent. The OS of the glioma patients was defined as the period from the day of surgery to death and the latest follow-up data were updated on December 31, 2019. This study was approved by the Institute Research Medical Ethics Committee of SYSUCC.

### Cell lines, plasmids, and regents

The cell lines U251, U373, A172, LNZ308, U138, T98G, and 293T and the normal astrocyte cell line (Ast) were used in our study. The cell lines U251, U373, LNZ308, U138, T98G, and 293T and the normal astrocyte cell line were obtained from the State Key Laboratory of Oncology in South China, and A172 cells were obtained from Dr Shing-shun Tony To, Department of Health Technology and Informatics, The Hong Kong Polytechnic University. U251, U373, A172, LNZ308, U138, T98G, and 293T cells were maintained in Dulbecco’s Modified Eagle Medium (DMEM) supplemented with 10% fetal bovine serum (FBS). Astrocytes were cultured in astrocyte medium (ScienCell Research Laboratories, Carlsbad, CA, USA). All these cell lines were authenticated by STR profiling within 6 months. All cells were maintained in a humidified incubator at 37 °C and 5% CO2. MG-132 and MK2206 were purchased from Selleck Chemical (Houston, TX, USA). CHX was purchased from MCE (Monmouth Junction, NJ, USA).

### RNA interference

For silencing of CBX3, predesigned siRNA oligonucleotides targeting CBX3 and control human nonsilencing siRNA were purchased from RiboBio Technology (Guangzhou, Guangdong, China). The transfections of siRNAs (75 nM) were performed using Lipofectamine 3000 (Invitrogen, Carlsbad, CA, USA).

### Lentivirus production and transduction

LINC00998 was stably silenced in U373 glioma cells using shRNA vectors based on the pLKO.1 plasmid (#10879, Addgene, Watertown, MA, USA). Two predesigned sequences targeting human LINC00998, along with a negative control sequence, were used. For LINC00998 overexpression, an H149 vector containing full-length LINC00998 was stably transfected into U251 and A172 cells using the VSVG, PLP1, and PLP2 lentivirus packing system according to the manufacturer’s protocol. Lipofectamine 3000 (Invitrogen, Carlsbad, CA, USA) reagent was used for transfection. Stably transfected cells were cultured in selection media supplemented with 2 μg/ml puromycin (MCE, Monmouth Junction, NJ, USA) for 2 weeks. Total RNA was isolated from U251, U373, and A172 glioma cells using an RNA Quick Purification kit (ESscience, Shanghai, China), and LINC00998 expression was determined by qRT-PCR using a Bio-Rad CFX96 Real-Time PCR System (Bio-Rad Laboratories, Inc., Hercules, CA, USA). All samples were analyzed in triplicate, and mRNA expression was normalized to that of the housekeeping gene *GAPDH* using the 2^−ΔΔCt^ method. The sequences of the primers are shown in Supplementary Table S1.

### Dual-luciferase reporter assay and IP assay

The 3′-UTR of LINC00998 (CACUGCC) and the MT form (GCGACGG) were separately subcloned into a pEZX-MT06 vector that contained the firefly luciferase gene (OBIO) to establish two constructs, namely, WT-LINC00998-luc and MT-LINC00998-luc, respectively. The pRL-CMV vector containing the Renilla luciferase gene acted as an internal control. Each overexpression construct, WT-LINC00998-luc or MT-LINC00998-luc, was cotransfected with miR-34c-5p mimics or control into U251 and A172 cells. Luciferase activities were detected 24 h after transfection by the Dual-Luciferase Reporter Assay System (Promega, Madison, WI, USA) according to the manufacturer’s instructions. For the IP assays, an anti-CBX3 (ab217999, Abcam, Cambridge, British) antibody was used, and the immunoprecipitation assays were performed using anti-ubiquitin (#3936, Cell Signaling Technologies (CST) Danvers, MA, USA) by western blotting.

### Subcellular fractionation analysis

Subcellular isolation of RNAs in A172 and U251 cells was conducted by the Cytoplasmic and Nuclear RNA Purification Kit (Norgen Biotek, Thorold, ON, Canada) according to the manufacturer’s instructions. Cytoplasmic and nuclear fractions were determined by qRT-PCR.

### Functional assays in vitro

Cell proliferation assays, colony formation assays, and sphere formation assays were used to evaluate the biological function of LINC00998 in glioma cells. Cell proliferation assays were performed as reported previously^35^. Briefly, 3000 cells were seeded in each well in 96-well plates. Cell viability was determined using Cell Counting Kit-8 (Dojindo, Kumamoto, Japan) according to the manufacturer’s instructions. Cell cycle analysis was performed with a cell cycle detection kit (Keygen, Nanjing, Jiangsu, China) according to the manufacturer’s instructions. The cells were then analyzed with an ACEA NovoCyte system (ACEA Biosciences, Inc., Santa Clara, CA, USA). For the colony formation assay, the transfected cells were seeded into six-well plates at 400 cells per well in DMEM supplemented with 10% FBS. After 14 days of culture, the culture was terminated when the colonies were visible. The culture medium was discarded before washing with PBS. The colonies were fixed with 4% paraformaldehyde for 30 min and stained with 0.1% crystal violet staining solution. Colony imaging was performed to determine the colony formation rate. For the sphere formation assay, 300 cells were seeded in ultralow attachment six-well plates in stem-like cell conditional medium (DMEM/F12, 20 ng/ml EGF, 20 ng/ml bFGF, 2% B27). Spheres with over 50 cells were counted after being cultured for 14 days. Each cell line was plated in triplicate, and all experiments were repeated at least three times. For cell apoptosis assay, briefly, cell apoptosis was detected using Annexin V–APC Detection Kit (Thermo Fisher), and analyzed using a CytoFLEX (Beckman Coulter Inc., CA, USA) flow cytometer.

### Xenograft growth of glioma cells in mice

Four-week-old female BALB/c nude mice were purchased from the Model Animal Research Center of Nanjing University (Nanjing, Jiangsu, China). Mice (six in each group) were randomly allocated to five groups. For the orthotopic animal model, U251 cells (4 × 10^5^ cells for U251 Vector and U251 LINC00998OE) and U373 cells (4 × 10^5^ cells for U373 ShCtrl, Sh#1-LINC00998, Sh#2-LINC00998) resuspended in PBS were intracranially injected 1 mm lateral and 2 mm posterior to the bregma and 4 mm from the surface of the skull using a microsyringe and 27 G needle as in our previous study^36^. Mice were observed every 3 days until moribund and then sacrificed. Another cohort (*n* = 1) was sacrificed 25 days post implantation. The brains were fixed, paraffin embedded, and sectioned for H&E staining. All animal studies were approved and performed by the animal institute of SYSUCC according to the protocols approved by the Medical Experimental Animal Care Commission of SYSUCC.

### LncRNA FISH and immunofluorescence staining

Subcellular localization of LINC00998 was determined in U251 and A172 cells using an RNA Fluorescence In Situ Hybridization Kit (Exonbio, Guangzhou, Guangdong, China) according to the manufacturer’s instructions. The expression level of LINC00998 in glioma tissue array was determined using In Situ Hybridization Kit (Exonbio, Guangzhou, Guangdong, China) according to the manufacturer’s instructions. The probe sequences are listed in Supplementary Table S1. The expression level of LINC00998 was determined by multiplying the area of the positive staining rates (0–100%) with the staining intensity (0, negative; 1, weak; 2, moderate; 3, intense). The scores were determined by two pathologists independently. The median score was chosen as the cut-off value for low and high expression. Hybridization with LINC00998 probes was first performed at 37 °C for 4 h, and then the probes were incubated with secondary antibodies protected from light for 1 h at room temperature. After rinsing three times with phosphate-buffered saline with Tween (PBST), DAPI-antifade (Exonbio) was added to stain the nucleus for 5 min. The images were obtained under a confocal microscope (Olympus, Tokyo, Japan).

### RIP assay and chromatin immunoprecipitation

RIP assays were performed using the RNA-Binding Protein Immunoprecipitation Kit (Millipore, Burlington, MA, USA) according to the manufacturer’s instructions. Five micrograms of rabbit anti-CBX3 (Abcam) antibody and five micrograms of rabbit anti-IgG (Millipore) were used to perform RIP. The enrichment of LINC00998 was determined by qRT-PCR. Chromatin immunoprecipitation (ChIP) assay were conducted according to the manufacturer’s instructions (Invitrogen) using PierceMagnetic ChIP Kit with anti-H3K9me3 antibody. Precipitated DNAs that contained c-Met fragments were then amplified using quantitative qRT-PCR. The primers of c-Met promoter used in ChIP assay were described in Supplementary Table S1.

### RNA pull-down assay and mass spectrometry

RNA pull-down assays were performed using the Pierce Magnetic RNA-Protein Pull-Down Kit (Thermo Fisher) according to the manufacturer’s instructions. Briefly, antisense and sense lncRNAs were transcribed in vitro by T7 RNA polymerase using a MEGAscript kit (Ambion, Carlsbad, CA, USA), and the product was labeled with biotin using a Pierce RNA 3′ End Desthiobiotinylation Kit (Thermo Fisher). Then, 50 pmol RNA was used for RNA pull-down. The eluted proteins were detected by western blot assay and analyzed by mass spectrometry (SAGENE, Guangzhou, Guangdong, China).

### Antibodies and western blot assay

Western blot analysis was performed using a standard protocol as reported previously^31^. Briefly, equal amounts of protein (30 μg) were separated via SDS-PAGE and electrotransferred onto polyvinylidene difluoride membranes (EMD Millipore). Membranes were probed with primary anti-CBX3 (ab217999, Abcam), anti-c-Met (ab51067, Abcam), anti-p-c-Met (ab68141, Abcam), anti-mTOR (#2983, CST), anti-p-mTOR (#5536, CST), anti-Akt (#4691, CST), anti-p-Akt (#4060, CST), anti-p70S6K (#2708, CST), anti-p-p70S6K (#9234, CST), anti-GAPDH (60004, Proteintech, IL, USA), anti-P27 (DF6090, Affinity, OH, USA), and anti-CDK1 (#9112, CST) and then probed sequentially with secondary goat anti-mouse and anti-rabbit antibodies (SA00001, Proteintech). The bound antibodies were visualized using an enhanced chemiluminescence kit (Beyotime, Shanghai, China).

### Statistical analysis

All data were analyzed using GraphPad Prism 8 (GraphPad Software, La Jolla, CA, USA) and are expressed as the mean ± standard deviation or standard error. Pearson’s correlation analysis was performed to determine the correlation between LINC00998 and CBX3 expression in the TCGA database. Differences were compared using Student’s *t* test for two groups and one-way ANOVA for multiple groups. The Kaplan–Meier method was used for survival analysis. *P* value < 0.05 was considered statistically significant.

## Supplementary information

Supplementary Figure Legends

Supplementary Figure 1

Supplementary Figure 2

Supplementary Figure 3

Supplementary Figure 4

Supplementary Figure 5

## Data Availability

The raw data of this paper have been uploaded onto the Research Data Deposit (RDD) with an RDD number of RDDB2020000884.
