# Supplementary material for: LncRNA LINC00998 inhibits the malignant glioma phenotype via the CBX3-mediated c-Met/Akt/mTOR axis
Source: Cell Death Dis. 2020 Dec 2;11(12):1032. doi: 10.1038/s41419-020-03247-6 (PMC7710718; doi:10.1038/s41419-020-03247-6)
Supplement: Supplementary file 1 — Supplementary Figure Legends [file 41419_2020_3247_MOESM1_ESM.pdf]

Supplementary Figure 1. The secondary structure and coding potential of LINC00998.

Supplementary Figure 2. LINC00998 induced apoptotic death in glioma cells.

Overexpression of LINC00998 increased apoptotic cells rate in both A172 and U251 glioma cells, while knockdown of LINC00998 decreased apoptotic cells rate in U373. (*p<0.05, **p<0.01, ***p<0.001)

Supplementary Figure 3. LINC00998 promoted tri-methylation of Histone H3K9.

A Western blotting showed that overexpression of LINC00998 increased H3K9me3 level while knockdown of LINC00998 decreased H3K9me3 level. (H3 used as the control) **B** Immunofluorescence results showed that overexpression of LINC00998 promoted H3K9me3 level.

Supplementary Figure 4. c-Met inhibitor exerts a similar effect as LINC00998 on glioma cells.

A, B A172 and U251 cell growth was inhibited gradually in a dose-dependent manner after SU11274 treatment at 1 µM and 5 µM. **C** The LINC00998 knockdown-promoted growth of U373 cells was reversed by SU11274 treatment at both 1 µM and 5 µM. **D, E** Clone

formation ability and sphere formation ability of A172 and U251 cells were inhibited gradually in a dose-dependent manner after SU11274 treatment at 1 μM and 5 μM. **F, G** The LINC00998 knockdown-promoted clone formation ability and sphere formation ability of U373 cells was reversed by SU11274 treatment at both 1 μM and 5 μM. (*$P < 0.05$, **$P < 0.01$, ***$P < 0.001$).

Supplementary Figure 5. mTOR inhibitor exerts a similar effect as LINC00998 on glioma cells.

A, B A172 and U251 cell growth was inhibited gradually in a dose-dependent manner after Rapamycin treatment at 1 μM and 5 μM. **C** The LINC00998 knockdown-promoted growth of U373 cells was reversed by Rapamycin treatment at both 1 μM and 5 μM. **D, E** Clone formation ability and sphere formation ability of A172 and U251 cells were inhibited gradually in a dose-dependent manner after Rapamycin treatment at 1 μM and 5 μM. **F, G** The LINC00998 knockdown-promoted clone formation ability and sphere formation ability of U373 cells was reversed by Rapamycin treatment at both 1 μM and 5 μM. (*$P < 0.05$, **$P < 0.01$, ***$P < 0.001$).

Supplementary Table 1. Sequences of primers and probes

LINC00998 F:5’- GTCACTGCTTTTGGTGCTGC-3’
LINC00998 R: -5’- ATATACCCCCAAGCAGGCAC-3’
U6 F: 5’-CTCGCTTCGGCAGCACA-3’
U6 R: 5’-AACGCTTCACGAATTTGCGT-3’
hGAPDH F: 5’- ACCACAGTCCATGCCATCAC -3’
hGAPDH R: 5’- CACCACCCTGTTGCTGTAGCC -3’
CBX3F: 5’-TAGATCGACGTGTAGTGAATGGG -3’
CBX3R: 5’-TGTCTGTGGCACCAATTATTCTT-3’
siCBX3#1: GGAGAATTGATGTTTCTCA
siLINC00998: GCCCTCAAGATTCCAACAT
siLINC00998: GGTTCCAAGAATCAGTAAA
c-Met promoter F: 5’-CCGCGGCGCCCCGAG-3’
c-Met promoter R: 5’-CGCCTCAGGGGTTCTGCT-3’

Supplementary Table 2. The proteins pull-downed by LINC00998

Protein	Score	Match	Match (sig)
RS27A	314	15	15
CBX3	276	9	9
H2A1	183	9	9
VIME	155	6	6
ACTB	131	6	6
H4	139	5	5
H2A3	125	8	8
ACTA	113	5	5
NPM	107	2	2
ATPB	106	5	5
ATPA	98	4	4
