# Supplementary figures and images for: LncRNA LINC00998 inhibits the malignant glioma phenotype via the CBX3-mediated c-Met/Akt/mTOR axis
Source: Cell Death Dis. 2020 Dec 2;11(12):1032. doi: 10.1038/s41419-020-03247-6 (PMC7710718; doi:10.1038/s41419-020-03247-6)

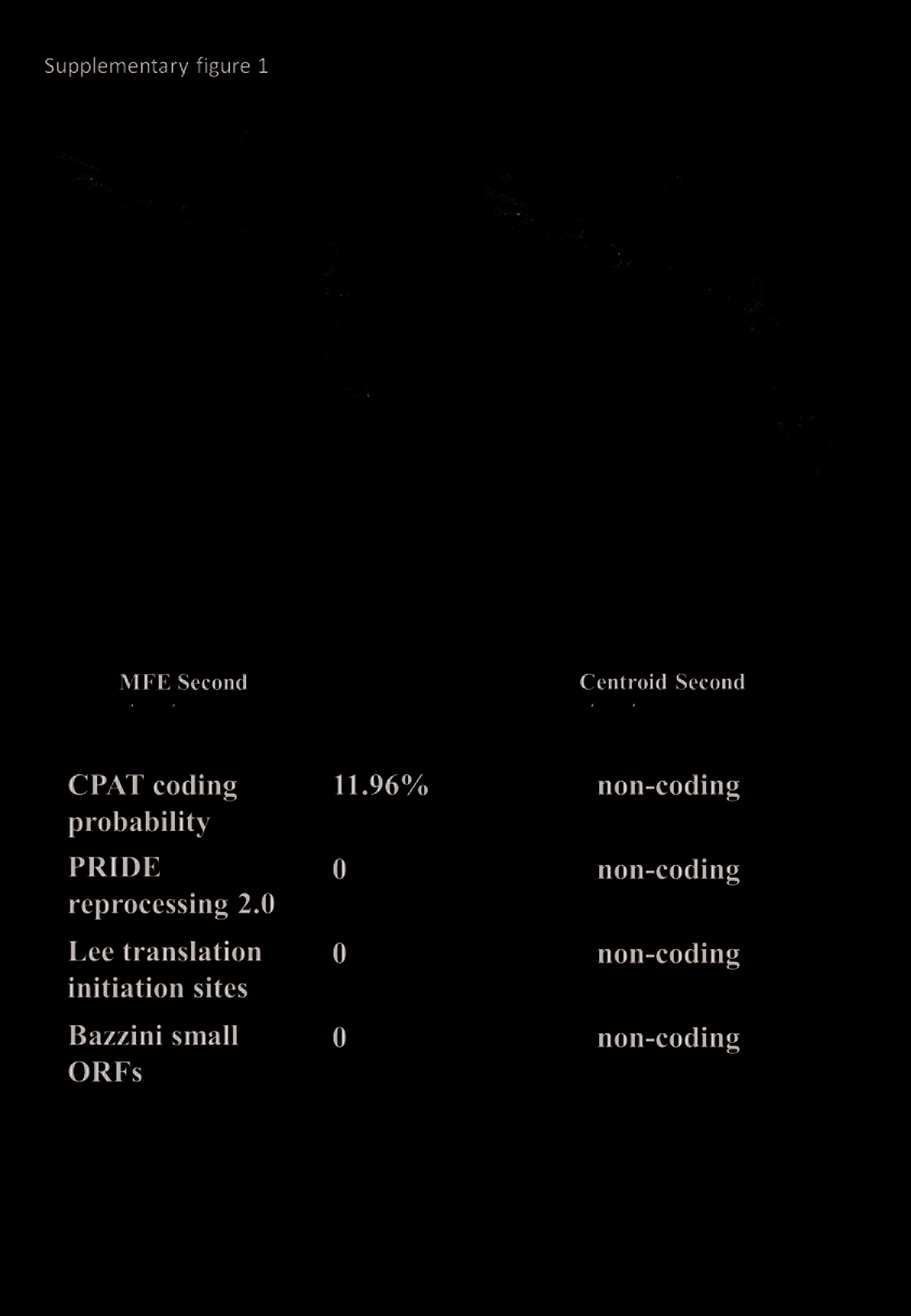

Supplement: Supplementary file 2 — Supplementary Figure 1 [file 41419_2020_3247_MOESM2_ESM.tif]

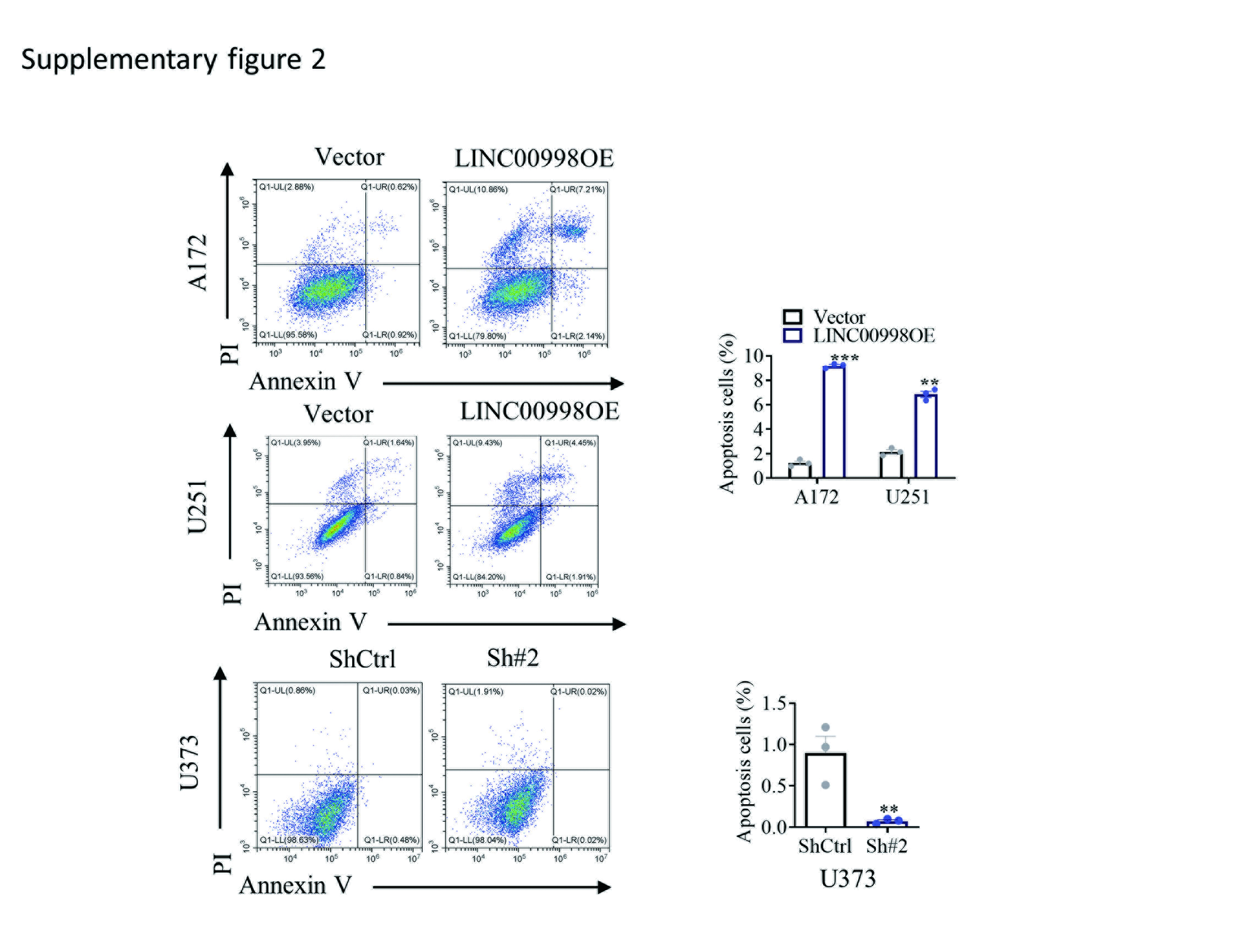

Supplement: Supplementary file 3 — Supplementary Figure 2 [file 41419_2020_3247_MOESM3_ESM.tif]

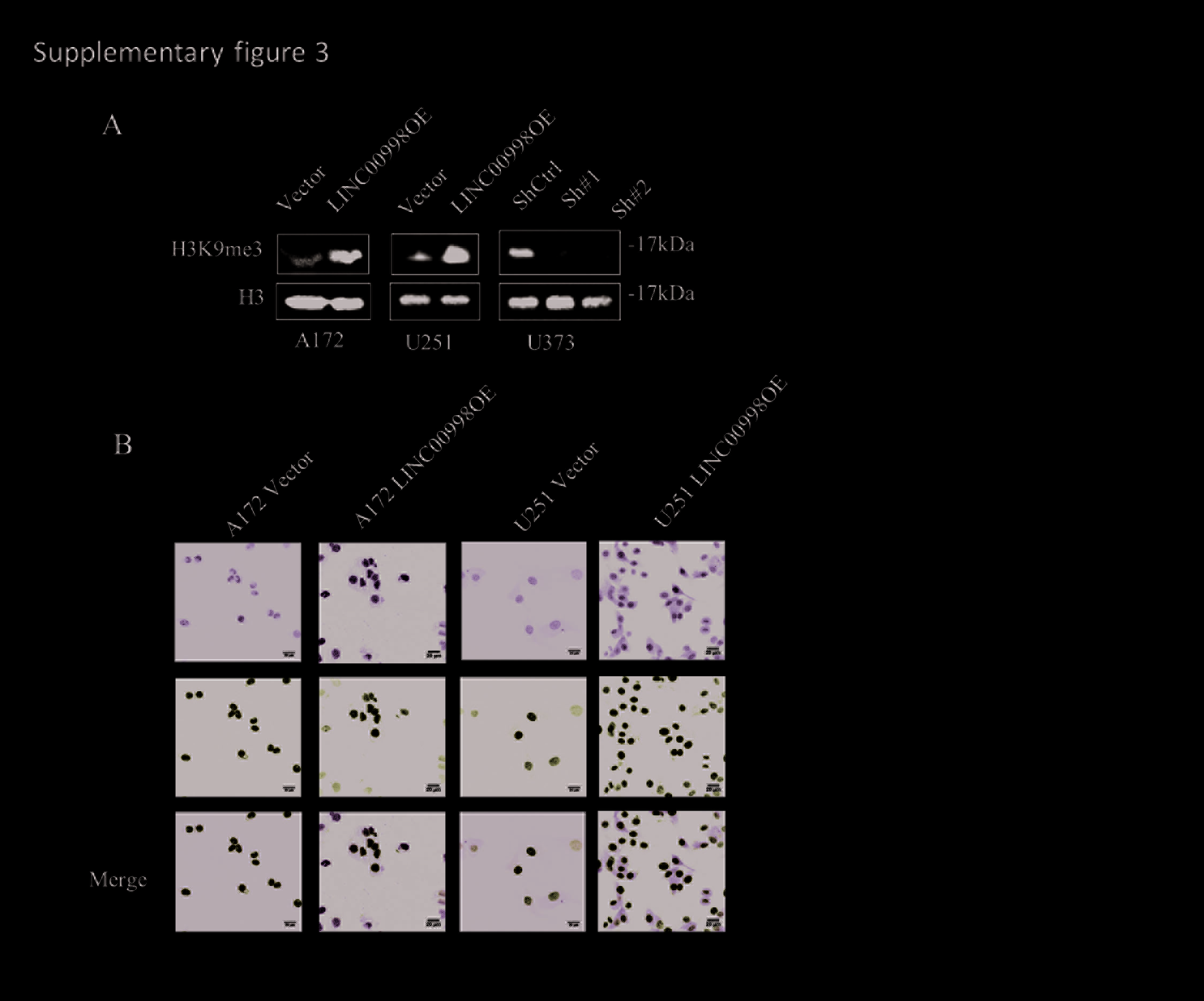

Supplement: Supplementary file 4 — Supplementary Figure 3 [file 41419_2020_3247_MOESM4_ESM.tif]

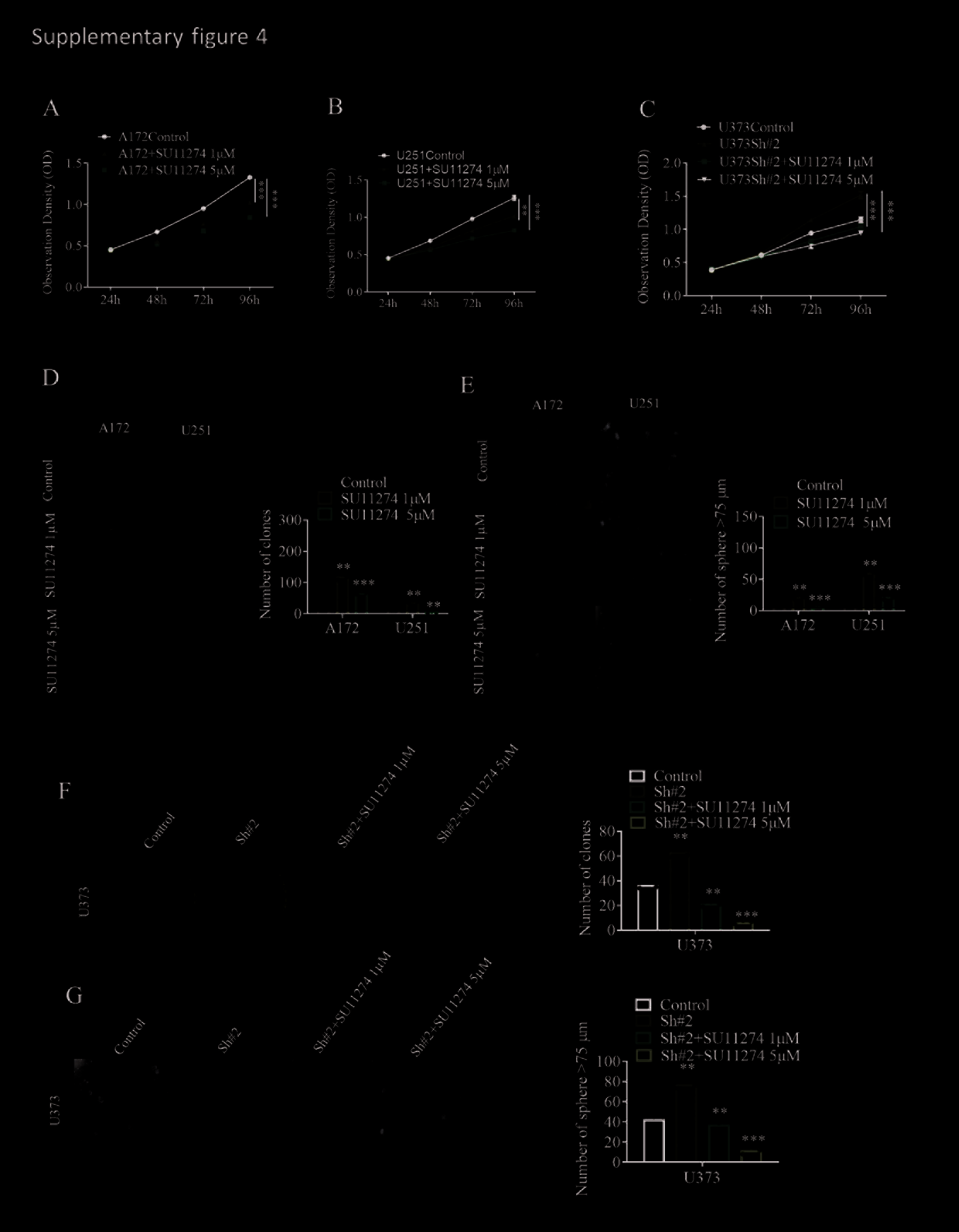

Supplement: Supplementary file 5 — Supplementary Figure 4 [file 41419_2020_3247_MOESM5_ESM.tif]

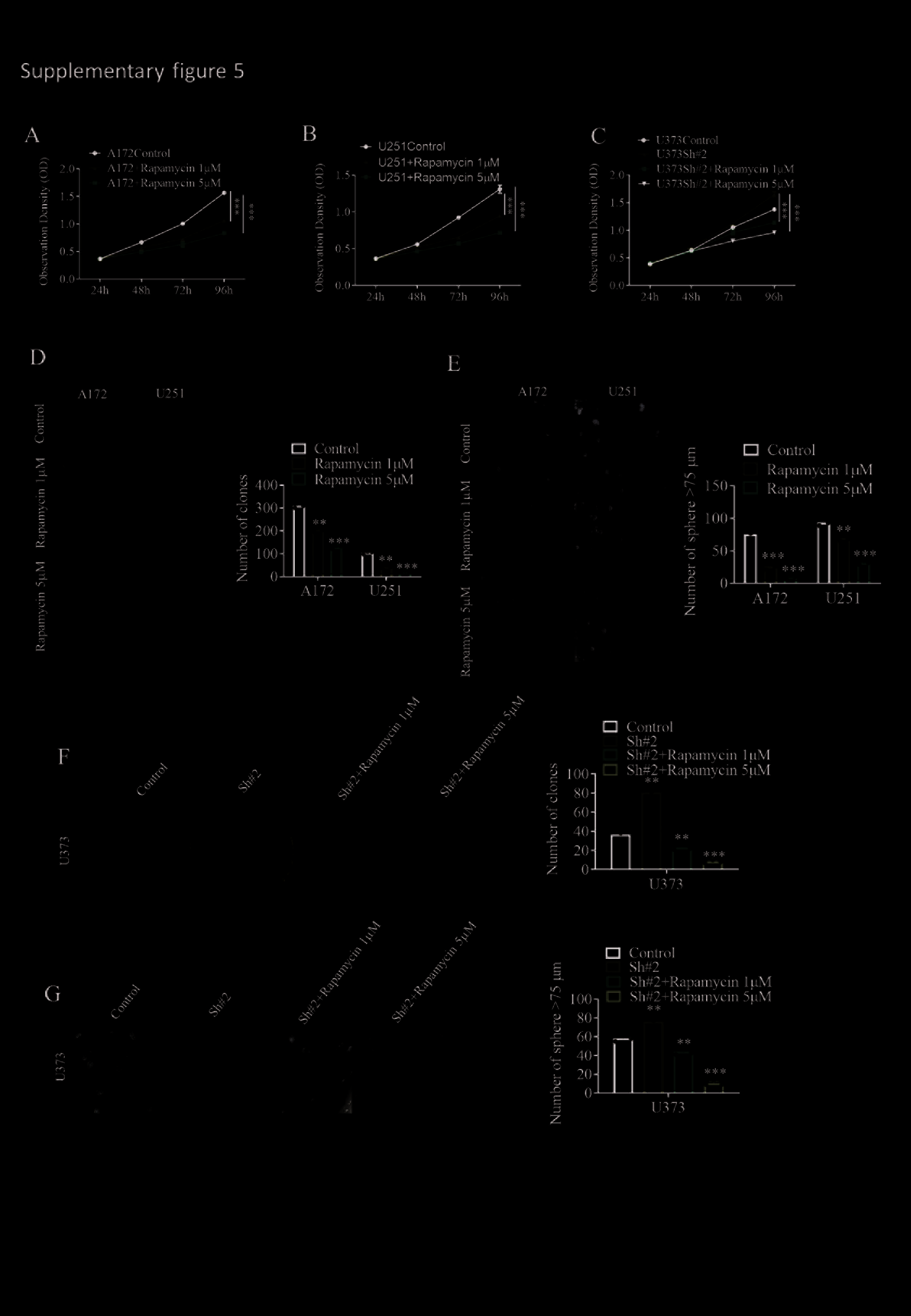

Supplement: Supplementary file 6 — Supplementary Figure 5 [file 41419_2020_3247_MOESM6_ESM.tif]
